# Comparative Physical–Mechanical Properties Assessment of Tailored Surface-Treated Carbon Fibres

**DOI:** 10.3390/ma13143136

**Published:** 2020-07-14

**Authors:** Dionisis Semitekolos, Aikaterini-Flora Trompeta, Iryna Husarova, Tamara Man’ko, Aleksandr Potapov, Olga Romenskaya, Yana Liang, Xiaoying Li, Mauro Giorcelli, Hanshan Dong, Alberto Tagliaferro, Costas A. Charitidis

**Affiliations:** 1 Research Lab of Advanced, Composite, Nanomaterials and Nanotechnology (R-NanoLab), Materials Science and Engineering Department, School of Chemical Engineering, National Technical University of Athens, 9 Heroon Polytechniou str., GR15780 Zographou, Greece; diosemi@chemeng.ntua.gr (D.S.); ktrompeta@chemeng.ntua.gr (A.-F.T.); 2 Yuzhnoye State Design Office, Krivorozhskaya Street 3, 49008 Dnipro, Ukraine; Ira.gusarova58@gmail.com (I.H.); Tamaramanko1607@gmail.com (T.M.); dnsk007@gmail.com (A.P.); Olgaromenskaja@gmail.com (O.R.); 3 School of Metallurgy and Materials, University of Birmingham, Birmingham B15 2SE, UK; YXL452@bham.ac.uk (Y.L.); X.LI.1@bham.ac.uk (X.L.); H.DONG.20@bham.ac.uk (H.D.); 4 Department of Applied Science and Technology (DISAT), Politecnico di Torino, 10129 Torino, Italy; mauro.giorcelli@polito.it (M.G.); alberto.tagliaferro@polito.it (A.T.)

**Keywords:** carbon fibres, electrochemical treatment, fibre/matrix bond, mechanical properties, physical properties, plasma treatment, surface properties, tailored properties, textile-reinforced composites

## Abstract

Carbon Fibres (CFs) are widely used in textile-reinforced composites for the construction of lightweight, durable structures. Since their inert surface does not allow effective bonding with the matrix material, the surface treatment of fibres is suggested to improve the adhesion between the two. In the present study, different surface modifications are compared in terms of the mechanical enhancement that they can offer to the fibres. Two main advanced technologies have been investigated; namely, plasma treatment and electrochemical treatment. Specifically, active screen plasma and low-pressure plasma were compared. Regarding the electrochemical modification, electrochemical oxidation and electropolymerisation of monomer solutions of acrylic and methacrylic acids, acrylonitrile and N-vinyl pyrrolidine were tested for HTA-40 CFs. In order to assess the effects of the surface treatments, the morphology, the physicochemical properties, as well as the mechanical integrity of the fibres were investigated. The CF surface and polymeric matrix interphase adhesion in composites were also analysed. The improvement of the carbon fibre’s physical–mechanical properties was evident for the case of the active screen plasma treatment and the electrochemical oxidation.

## 1. Introduction

Carbon fibres (CFs) are used as reinforcement for polymer composites, being among prospective materials in an aerospace domain [1]. An increased focus on carbon fibre-reinforced polymers (CFRPs) is specified by a unique set of their technically valuable properties, such as their high specific strength, low tensile strength at deformation, high thermal stability and conductivity. The main requirements to expand their applications are to enhance their mechanical properties while maintaining cost effectiveness of manufacturing process and final cost of the product.

The physical and mechanical characteristics of composite materials directly depend on the adhesion strength of the CF surface and polymeric matrix interphase [2]. As a result of the significant price of high-quality CFs, their application in various industries is currently not affordable [3]. CFs are usually manufactured from polyacrylonitrile, sinters or viscose precursor. The heat treatment of fibres obtained based on polyacrylonitrile under temperature higher than 1000 °C leads to almost full carbonization, as well as to more structured graphite microstructure and increased modulus of elongation [4]. The estimated cost of CF manufacturing with modulus of 270 GPa is twice higher than for fibres with modulus of 220 GPa. Improving the mechanical properties of CFs obtained from cost-effective raw materials will reduce the cost of the end product and expand the CFRPs market [5]. 

CFs have lots of surface defects, formed as a result of the production technology selected by industries, namely, the thermo-oxidizing method [6]. Selected treatments affect the roughness, surface morphology, and the porosity of the fibres [7]. During manufacturing, CFs accumulate defects, so the actual strength is significantly lower than the theoretical. To enhance the CF strength, carbon nanostructures, such as particles of nanographite, multi- and single-wall carbon nano-tubes, fullerenes and fullerene-like structures as modifying dopant for fibres have been investigated [8]. There are also techniques related to the doping of carbon nanotubes into the precursor, but these methods require complicated and expensive processes and equipment [9]. Another method with more feasibility is CF reinforcement using two-phase heat treatment [10]. However, with this method micro-stresses are removed, while micro-cracks remain in the material [11].

The adhesion strength can be increased by the formation of oxygen-containing active groups through surface modification that can create covalent bonds with the active groups of resin leading to a composite material with improved interfacial properties [12]. The main issue is that, during the modification of fibres, the sintering out of fibre pores, micro-cracks, and fibre destruction can occur [13]. In this regard, in order to select surface treatment methods and modes, which do not evoke fibre destruction, it is required to investigate their effect on single fibre strength.

Surface treatments (continuous or batch type) can be categorized as: (1) oxidative and (2) non-oxidative treatments [14]. The oxidative treatments can be further subdivided into: (a) low-phase gas oxidations; (b) liquid phase oxidations carried out chemically or electrochemically; (c) catalytic oxidations. Non-oxidizing treatments that improve fibre–resin adhesion involve: (a) the deposition of more active carbon forms such as whiskers, pyrolytic carbon or (b) polymer grafting on the surface of carbon fibres [15].

The oxidative treatment of a carbon material results in [16]:

- Ion-exchange properties to the material;
- Increases sorption capacity by developing porous structure and surface;
- Increases mechanical strength;
- Increases adhesion to polymers and inorganic binders.

The increase in carbon fibre strength by oxidation is based on the fact that oxidizing agent initially reacts with defective areas of the fibres, either by healing the imperfections by forming intermolecular bonds or by eroding the defects. Acid surface groups play an important role in the fibre/matrix interphase by: (1) forming chemical bonds with the matrix molecules, (2) improving the wettability of the fibre and (3) increasing the surface area of the fibre [17]. As result of the plasma treatment on carbon fibre surface, the reaction capacity between fibre and matrix surface grows due to the increase in the amount of COOH, –C–OH and =C=O groups onto the fibre surface [18].

According to the above, plasma treatment seems a novel and promising method of fibre surface modification of the chemical and physical structure of CF’s external layers. The main advantages and novelty of plasma treatment application onto CFs are:

- The possibility of process optimization, thanks to the wide range of controllable parameters;
- The environmental safety, since it does not form chemical wastes;
- The wide variety of gases, which can be used in such applications.

Lately, a new type of plasma treatment, active screen plasma (ASP), is being developed and discussed in this study, being of undisputable interest for modification of CFs surface. One of the main advantages of ASP process is floating potential of components, which allows nitrogenization and carbonization of insulating materials [19]. Moreover, ASP is compared to low-pressure plasma (LPP) in terms of the CFs properties tailoring. Specifically, the analysis of the effect on HTA-40 carbon fibre strength properties, due to their surface modification by electrochemical treatment, as well as by the two plasma treatments, is presented in this study, utilising advanced characterisation techniques such as single fibre testing.

## 2. Materials and Methods 

### 2.1. Electrochemical Treatment

Surface treatment of Tenax HTA-40 (Teijin, Tokio, Japan) carbon fibre was conducted in two stages, starting with an oxidative treatment (electrochemical oxidation) that is followed by a non-oxidative (electropolymerisation). The electrochemical oxidation was conducted via cyclic voltammetry to increase carbon fibre surface roughness and formation of active centres [16]. The fibres surface activation was followed by their electropolymerisation via chronoamperometry in solutions of different monomers: acrylic acid (polymer PAA), methacrylic acid (polymer PMAA), acrylonitrile (polymer PAN) and N-vinyl pyrrolidone (polymer PVP), as presented in [20]. All monomers were purchased from Acros Organics (Waltham, MA, USA) with a purity of 99%. Basic characteristics of HTA-40 CF can be found on Table 1. 

#### 2.1.1. Electrochemical Oxidation

Electrochemical conditions were selected, considering the existing experience of PAN-based commercial carbon fibre treatment, which are focused on formation of oxygen-containing groups with high surface concentration [21]. Cyclic voltammetry was conducted in each specimen with multiple sweeps (5, 10, 15, 20) in the region of −3 V to 3 V (scan rate 100 mV/s), in a 5% solution of H_2_SO_4_ purchased from Fisher Scientific (purity 95%). Comparing the tensile strength of the pristine and modified single CFs is one of the effective ways to assess the effect of the fibre treatment process on its mechanical properties [22]. To perform trade off analysis of different treatment methods effect on carbon fibre strength, the tensile stress at break of single fibres was determined. Regarding sample manufacturing, single CFs with length of 30 mm, selected from tows of the investigated materials, were pasted into mounting frame, providing its coaxial fixing in testing machine. Tensile strength tests were carried out at 20 samples from each batch.

#### 2.1.2. Electropolymerisation

Since under cyclic voltammetry, fibre surface destruction is favoured with the increase in the number of scanning cycles, the following process was selected for fibres’ treatment:

1. Electrochemical oxidation in 5% aqueous H_2_SO_4_ solution during 10 cycles of potential changes in a range of −3 V to +3 V with the velocity of 100 mV/s;
2. Electropolymerisation in potentiostatic conditions.

Vinyl monomers, according to Gubanov ’s work [8], are well studied as carbon fibres coating. In this regard, four different monomers were selected, in particular, acrylic acid (polymer PAA), methacrylic acid (polymer PMAA), acrylonitrile (polymer PAN) and N-vinyl pyrrolidone (polymer PVP), as presented in our previous work in [22]. Electropolymerisation was conducted under room temperature in a 150-mL single-chamber electrochemical cell using three electrode system. After the finishing of treatment, the fibres were washed with water and acetone, and dried in a furnace. 

### 2.2. Plasma Treatment

#### 2.2.1. Low-Pressure Plasma

**Figure 1:**
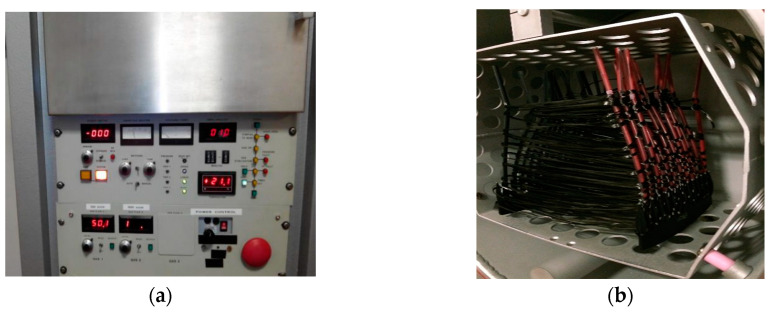
LPP (**a**) control panel and (**b**) chamber.

The effect of LPP on modification of carbon fibres surface was studied in order to improve interphase fibre adhesion to epoxy matrix in composites. The carbon fibre was rolled on a frame continuously to a length of 2 m, as shown in Figure 1. Their surface was modified by LPP under the pressure, range between 1–100 Pa. HTA-40 carbon fibre LPP treatment modes are presented in Table 2. 

#### 2.2.2. Active Screen Plasma

**Figure 2:**
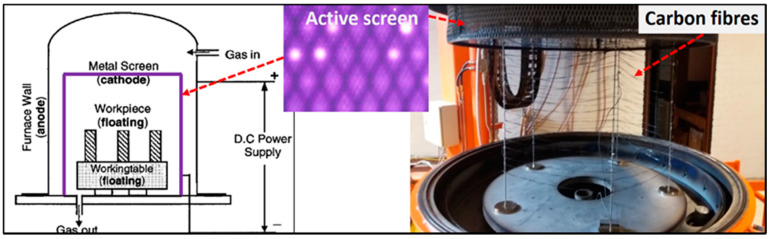
Schematics (left) and experimental chamber (right) of ASP treatment on CFs.

Under ASP treatment, a cathode is connected to a metallic screen, installed around the working table, to which floating potential is applied. During ASP treatment, the plasma forms on the metallic screen surface, and the carbon fibres set on the working table are post discharged (Figure 2) with ions, electrons and radicals [18].

ASP treatments were carried out in a Plasma Metal 75 kVA + 15 KV industrial scale unit at pressures of 20–110 Pa. The voltage controlled from 250 to 350 V was applied between the active screen (cathode) and the wall of the furnace (anode) during all processes. The CF tows were hung on a set of stainless-steel bars with a distance of 15 cm to the active screen (Figure 2 right). The treatment current was recorded between 60 and 70 A, and the temperature in the furnace was recorded between 17 to 95 °C. 

### 2.3. Characterisation Methods

Scanning electron microscopy (SEM, JEOL 7000, Oxford Instruments, Oxfordshire, UK) were used to observe the changes in the surface morphologies of CFs. X-ray photoelectronic spectroscopy (XPS),) analyses were performed with XPS versa Probe 5000 (PHI Electronics, Chigasaki, Kanagawa, Japan).

The single carbon fibre tensile tests samples were selected from the tows of treated materials and cut into 30 mm length. They were pasted into a mounting frame, providing its coaxial fixing in testing machine. Tensile strength tests were carried out at 20 samples for each batch. The fibre’s tensile strength was determined at samples of micro-plastic according to ISO 10618 “Carbon fibre—Determination of tensile properties of resin-impregnated yarn” standard. Micro-plastic samples were manufactured from carbon binder, impregnated with liquid Huntsman epoxy resin, at MAW 20 FB5/1 winding machine (Mikrosam AD, Prilep, North Macedonia). After winding, the samples were cured in furnace. Breaking load was monitored using the testing machine dynamometer (Universal Testing Machine, Instron, Norwood, MA, USA).

## 3. Results and Discussion

### 3.1. Assessment of Electrochemical Treatment

**Figure 3:**
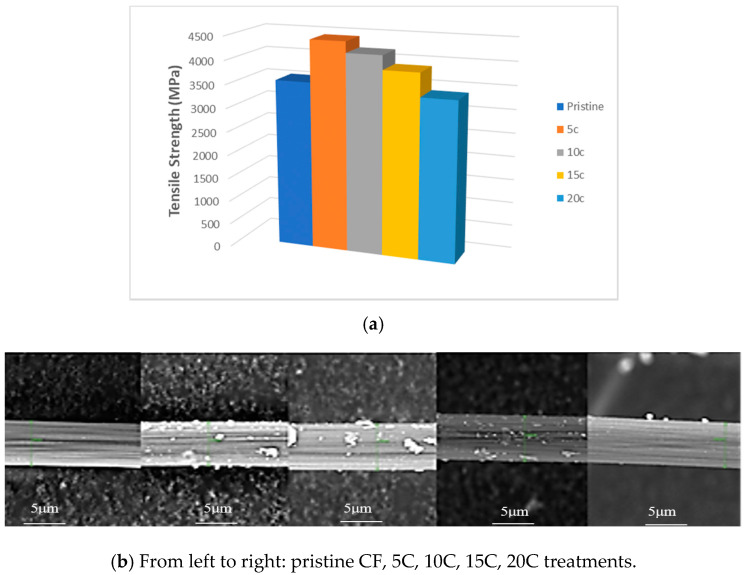
Effect of electro-chemical treatment on single fibres strength (**a**) and microstructure (**b**).

After the cyclic voltammetry, an enhancement of tensile strength was noticed (Figure 3a). The maximum tensile strength value of 4433 MPa, exceeding the strength of pristine fibres by 20%, was achieved under five cycles of treatment. The ultimate tensile stress value decreases with the increase of treatment cycles (up to 20). Twenty cycles of treatment lead to strength reduction by 4%. HTA-40 carbon fibre strength changing is not in conflict with the results of its surface microstructure studies. Based on microstructural analysis, it was discovered that foreign inclusions emerge at their surface, which are not present in the pristine state material (Figure 3b). 

**Figure 4:**
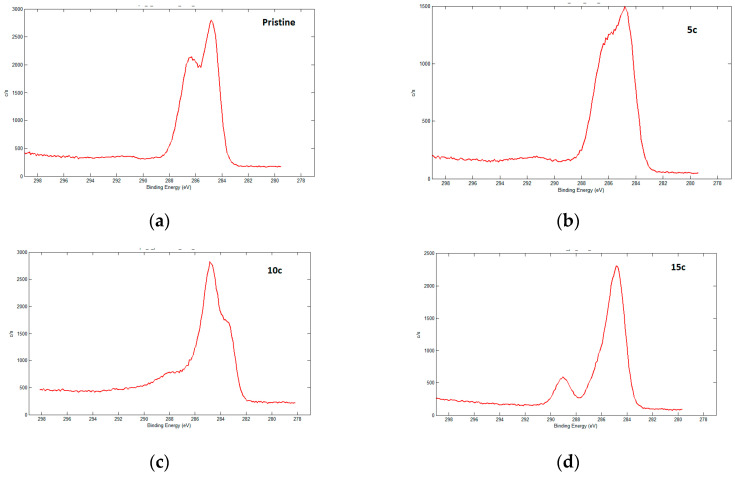
High-Resolution XPS spectra of C1s for (**a**) Pristine CF, (**b**) after 5, (**c**) 10 and (**d**) 15 cycles of electrochemical treatment.

After 5–15 cycles of electrochemical oxidation, a lot of new build-ups are formed at the fibre surface; however, after 20 cycles their number significantly reduces. Probably, this can be classified as external layer structure changing and removal of new build-ups from the surface, which lead to carbon fibre destruction [23]. Using X-ray photoelectronic spectroscopy, it was discovered that—despite the formation of active oxygen-containing groups at the carbon fibre surface under 5–15 cycles of electrochemical oxidation— the chemical compounds between the fibres oxidized by the electrochemical method and epoxy resin are not key for defining interphase strength in composites (Figure 4) [13]. The main reason for that is the low concentration of functional groups at the surface [24]. Therefore, cyclic voltammetry was applied as a stage of surface activation for further modification via electropolymerisation.

**Figure 5:**
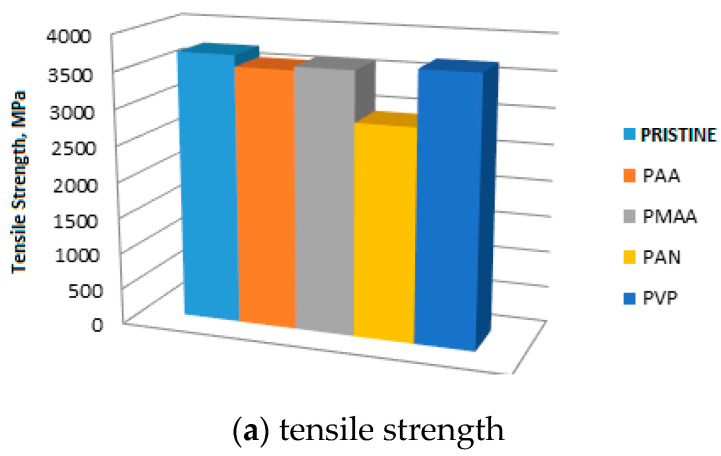

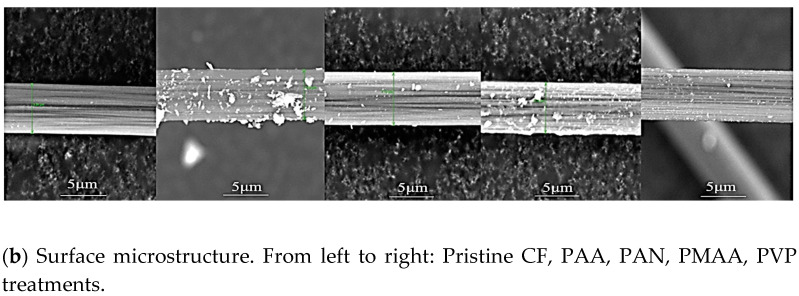
HTA-40 fibre tensile strength (**a**) and surface microstructure after electropolymerisation in solutions of different monomers (**b**).

**Figure 6:**
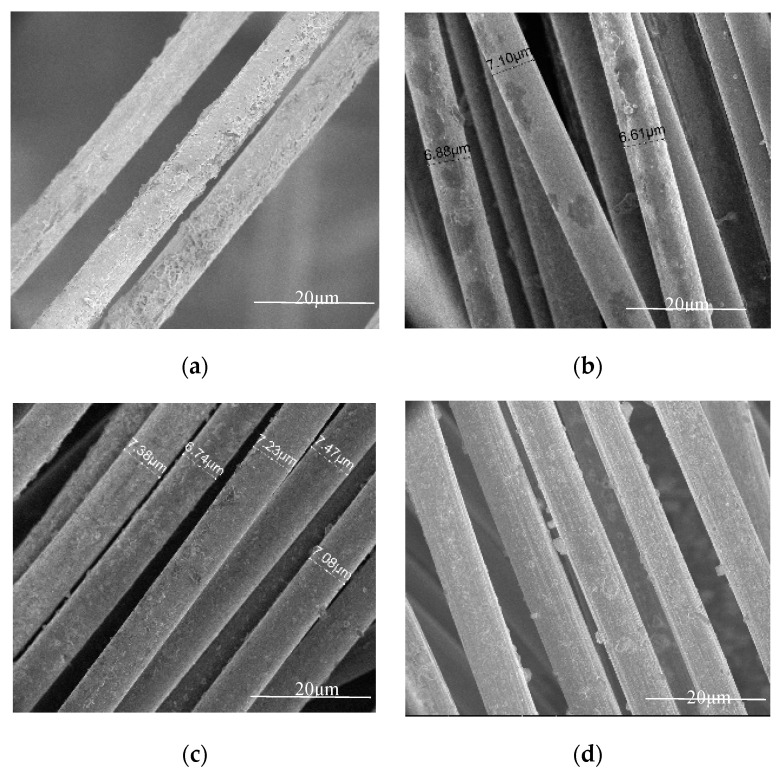
Fibres after polymerisation: (**a**) HTA-40 with PAA, (**b**) HTA-40 with PMAA, (**c**) HTA-40 with PAN, (**d**) HTA-40 with PVP.

To study the effect of electropolymerisation on HTA-40 carbon fibre properties, the tensile strength of single fibres and their surface microstructure in pristine state and after treatment at the electronic scanning microscope were determined (Figure 5 and Figure 6).

Fibres after electropolymerisation in PMAA and PVP solutions have a tensile strength at the level of pristine fibre, 3600 MPa. A 20% strength reduction of fibres was noticed after treatment in PAN solution. During the investigation of the HTA-40 carbon fibre microstructure after electropolymerisation, it was noticed that in all monomer solutions formation of the corresponding polymeric coatings at the fibre surface occurred, e.g., the new build-ups at the fibre’s surface were observed. Maximum amount of polymer was noticed under PAA treatment. The results of microstructural analysis are presented in Figure 6. Under electropolymerisation, polymer forms at the surface of HTA-40 carbon fibre, which can heal defects and enhance its strength.

It should be noted that, according to the literature [25] and observation of its surface, the polymerized acrylonitrile formed into pellets, exhibiting crystalline polymer properties associated with a unique two-dimensional order. Moreover, the observed crystalline structures could be attributed to the residual electrolyte (ZnCl_2_) molecules that tend to form hexagonal crystal-like deposits which demonstrate some properties of crystalline polymer, resulting from its planar order [26]. After treatment in the solution of acrylic acid, similar build-ups are formed, with the polymer covering the entire surface of fibres. After treatment with PMAA and PVP, a smooth polymeric coating is formed at the fibre’s surface.

**Figure 7:**
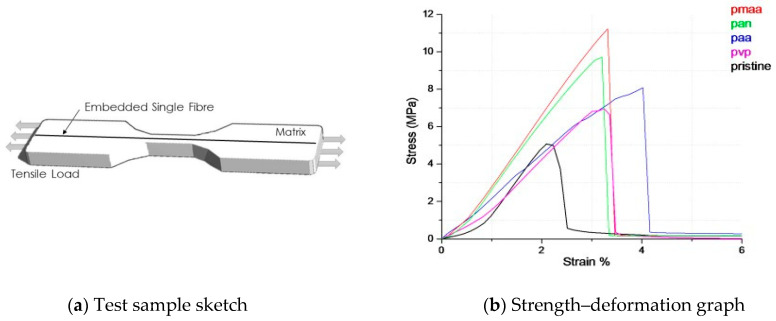
Test Sample Schematic (**a**), and correlation of strength properties of composites with single fibre after electropolymerisation with different monomers (**b**).

To estimate the mechanical properties of composites with single fibre after electropolymerisation in solutions of different monomers, the stress–deformation graphs of specifically made samples were obtained (Figure 7). 

Based on the obtained data it was found that strength of composite with carbon fibre after electropolymerisation in all analysed monomer solutions was in the range of 67–112 MPa, which exceeds the strength of samples with pristine fibre of 50 MPa. The maximum strength of 112 MPa was achieved after PMAA, followed by 97 MPa of PAN, 81 MPa of PAA and finally 67 MPa of PVP.

The samples after PAA show the highest value of elongation, which is 4%, probably due to the heavier molecular weight of polymer in comparison with others. The modulus of elasticity of samples with pristine fibre is 3.7 GPa. After PMAA treatment, it remained practically at the same level (3.8 GPa). All other types of polymerisation led to a reduction of the pristine fibre modulus of elasticity to 2.5–3.5 GPa. According to the literature [27], the thickness of the interlayer that is formed through electropolymerisation between the carbon fibre and the matrix is crucial to the tensile and flexural properties of the composite. The size of grafted material is inversely proportional to the modulus of the material. The results of the conducted experimental studies showed that the most prospective treatment is PMAA, since it leads to maximum properties improvement of composite with treated carbon fibre. 

### 3.2. Assessment of Low-Pressure Plasma Treatments

**Figure 8:**
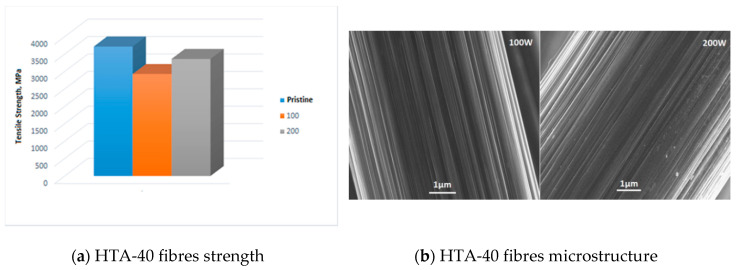
Dependence of (**a**) HTA-40 fibres tensile strength and (**b**) microstructure on LPP treatment modes.

**Figure 9:**
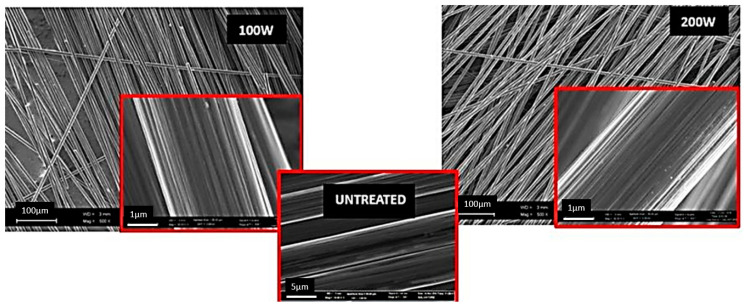
Microstructure of HTA-40 fibres before and after LPP treatment.

Studying of single fibres strength after treatment with oxygen plasma with the capacity of 100 W and 200 W during 5 min led to reduction of their strength by 10–15% in comparison with untreated fibre (Figure 6a). However, during microstructural analysis of HTA-40 fibre no visible defects, inclusions or structural damages of fibres were found (Figure 8b and Figure 9).

Variations in the surface properties after LPP treatment were, as evidenced by the intensity of its electron reflection capability, observed in image tone density (Figure 9). Untreated fibres have a dark tint, while during LPP treatment with the capacity of 100W they become lighter and, finally, during LPP treatment with capacity of 200 W, they acquire the lightest tint. Defects at the surface are not observed. 

Consequently, under the effect of LPP, a surface modification of carbon fibres is observed, while their strength reduces. The obtained results correspond to the data in the literature. Plasma treatment of carbon fibres within the timeframe of 2–30 min leads to a reduction of ultimate tensile strength by 8% [28,29]. 

### 3.3. Accesment of Active Screen Plasma Treatments

**Figure 10:**
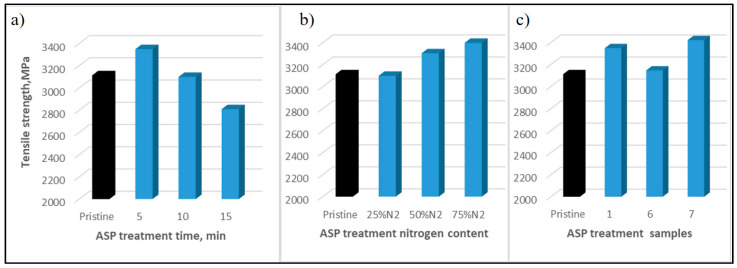
Tensile strength of ASP-treated carbon fibres compared with pristine ones: (**a**) under atmosphere of 25%N_2_ + 75%H_2_ with different treatment times; (**b**) same treatment time of 10 min with different nitrogen content; and (**c**) same treatment time of 5 min with different conditions, as indicated in Table 3.

A set of active screen plasma (ASP) treatments under different treatment conditions in terms of gas mixture, pressure and time were carried out, and the details are listed in Table 3. A large number of single fibre tensile tests were carried out and the results are shown in Table 3. Figure 10a,b compared the treated CFs with pristine ones in two groups: (a) under atmosphere of 25%N_2_ + 75%H_2_ with different treatment time; (b) same treatment time of 10 min with different nitrogen content; and c) same treatment time of 5 min and pressure but different gas mixture. It can be seen that the tensile strength of ASP treated carbon fibres are higher for shorter time treated CFs than longer time ones. The tensile strength of 5-min-treated CFs are increased 8% from pristine ones. However, the 15-minute-treated CFs reduced the strength to 2809 MPa, nearly 10% lower than the pristine ones. While changing the ASP treatments gas ratio of H_2_ and N_2_ and keeping other treatment conditions the same with the 10 min time, the tensile strength shows proportional relationship to the N_2_ contents, but the strength of lowest nitrogen content of 25%N_2_ treated CFs is similar to the pristine ones. When the treatment time duration was 5 min with the same treatment pressure, the tensile strengths of the treated CFs are all higher than the pristine ones and the CFs treated with gas mixture of H_2_ and Ar are shown strength increase in big extent.

**Figure 11:**
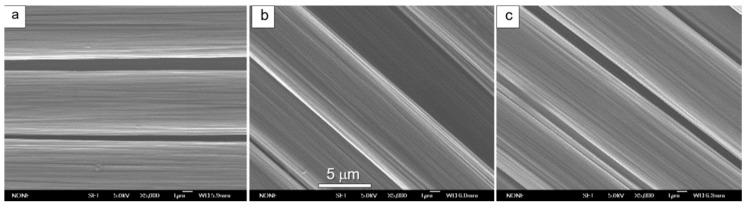
SEM images of HTA-40 carbon fibres: (**a**) as received; (**b**) ASP1 and (**c**) ASP2 treated (conditions shown in Table 3).

Based on the outcomes of the single fibre tensile strength and the other characterisations [18], two newly designed treatment conditions (Table 4)—modified from the conditions of sample number 1 and 7, shown in Table 3—were set up for ASP treatments on the carbon fibres. Both ASP treatments kept a constant pressure (75 Pa), time (5 min) and power (~23 KW) but with different gas mixture compositions: ASP1 = 25% N_2_ + 75% H_2_ and ASP2 = 5% Ar + 23.75% N_2_ + 71.25% H_2_. The surface morphology of the newly treated and pristine CFs were observed by SEM. As can be seen from Figure 11, the surface of all fibres presented similar ridges and striations parallel to the fibre axial direction owing to the PAN manufacturing process. No noticeable surface damage can be observed after the plasma treatments, indicating limited bombardment of the ions to the carbon fibre surfaces from the active screen plasma treatment comparing to the conventional DC plasma treatments.

Carbon fibre-reinforced polymer composite samples were made with ASP1- and ASP2-treated CFs (conditions shown in Table 3) and the pristine ones for tensile strength, interlaminar shear and bending strength evaluation. The unidirectional carbon composite samples were produced by filament winding method and the epoxy used is the LY556 resin system from Huntsman. Table 4 listed the strengths of the carbon fibre-reinforced polymer (CFRP) composite samples made with pristine and treated carbon fibres under modified active screen plasma (ASP) treatment conditions. The tensile strength results tested from the microplastic composite samples show the ultimate tensile strength of pristine and ASP1 treated CFs possess comparable values, 3420 and 3398 MPa, respectively. Composites made with the fibres treated with the ASP2 condition shows reduced strength of 3117 MPa, which is 9% lower than that of the pristine ones. The interface adhesion strength of the composites is reflected from the shear and bending tests results shown in Table 4. It can be seen that the ASP treatments have shown a moderate effect on the shear strength under current test conditions, in the order of a 3% variation. However, the bending strength of ASP1 treated CFRP composite were enhanced from 872 and 65 MPa for pristine ones both along and across the fibre’s axis to 1015 and 76 MPa, respectively, a 16% increase of the bending strength. With ASP2 treated CFRP composite, the bending strength along fibres is enhanced by 11%, but a decrease by 5% across CF axis.

As a result of a strengths evaluation, ASP1-treated HTA-40 fibres revealed higher values of strength characteristics both in fibres and in composite samples than that of the pristine ones. As such, 9000-m-long ASP1-treated CFs have been validated by making airspace components of propellant tanks (by one of the co-authors, Yuzhnoye). The outcomes of the evaluation showed a 12% improvement in the strength of the tanks made from the modified CFs than the ones made from the pristine CFs.

## 4. Conclusions

Experimental studies of modifying the effect on HTA-40 carbon fibre surface and the physical–mechanical properties of pristine fibres were performed for the first time by means of cyclic voltammetry, electropolymerization in acrylic, methacrylic acids monomers, acrylonitrile, N-vinyl pyrrolidone media, as well as low-pressure plasma and active screen plasma treatments.

It was determined that, under cyclic voltammetry, the maximum value of tensile strength, 4433 MPa, exceeding pristine fibres strength by 20%, was achieved in under five cycles of treatment. An increase in the treatment cycles number results in decreasing the breaking stress value. The electropolymerization in PMAA and PVP solutions does not affect the tensile strength of pristine fibre. A decreasing of the fibre’s strength by 20% was noted after treatment in the PAN solution. Microstructural studies proved the formation of the corresponding polymeric coating at the fibre surface. The most prospective polymer coating is the PMAA, since it leads to maximum composite properties with treated carbon fibres (an increase of 124% in tensile strength), and does not cause a reduction of the mechanical properties of the fibre itself, which comes in accordance with our recent studies [30,31]. Under the modification of carbon fibres by low-pressure plasma treatment, CF’s tensile strength is reduced, while under modification by active screen plasma, all five-minute-treated CFs show increased tensile strength.

Hence, in order to improve physical–mechanical characteristics of CFRPs, it is reasonable to modify CFs using cyclic voltammetry in a solution of 5% sulfuric acid under five cycles with further PMAA treatment and an active screen plasma treatment of ASP1. From this study, it was evidenced that the properties of CFs and subsequently the performance of CFRPs can be tailored after specific oxidised (electrochemical treatment and plasma) or non-oxidised (electropolymerisation) CF surface treatments.

## Figures and Tables

**Table 1:** HTA-40 CF specifications.

CF	Number of Filaments	Nominal Linear Density	Tensile Strength (MPa)	Tensile Modulus (GPa)	Elongation at Break (%)	Filament Diameter (μm)	Density (g/cm^3^)	Sizing Type
HΤA 40 Ε13	6000	400 tex	3950	238	1.7	7	1.76	Epoxy 1.3% w/w

**Table 2:** Carbon fibre LPP treatment modes.

Item Number	Reagent	Treatment Time (min)	Capacity (W·s)	Pressure (Pa)	Gas Concentration
1	O_2_	5	100	20–40	75
2	O_2_	5	200	20–40	75

**Table 3:** Active screen plasma (ASP) treatments conditions for carbon fibres and their single fibre tensile strengths.

Sample	Atmosphere	Time (min)	Pressure, Pa (±20)	Tensile Strength, MPa
Pristine	-	-	-	3113
1	25%N_2_ + 75%H_2_	5	70	3349
2	25%N_2_ + 75%H_2_	10	70	3099
3	25%N_2_ + 75%H_2_	15	70	2809
4	50%N_2_ + 50%H_2_	10	70	3304
5	75%N_2_ + 25%H_2_	10	70	3399
6	25%Ar + 75%H_2_	5	70	3147
7	20%Ar + 80%H_2_	5	70	3422
8	25%N_2_ + 75%H_2_	10	50	3755

**Table 4:** Strengths of the CFRP composite samples made with pristine and treated carbon fibres under modified active screen plasma (ASP) treatment conditions.

HTA-40 Carbon Fibre Treatment	Pristine	ASP1 Treatment (75%H_2_ + 25%N_2_, 5 min, 75 Pa)	ASP2 Treatment (71%H_2_ + 5%Ar + 24%N_2_, 5 min, 75 Pa)
Breaking tensile strength, MPa	3420.0	3398.0	3117.0
Breaking strength under shearing, MPa	66.0	68.3	63.9
Breaking strength under bending, (along/cross fibres), MPa	872.1/65.2	1015.01/76.4	972.8/61.6

## References

[1] Tiwari S., Bijwe J. (2014). Surface Treatment of Carbon Fibres–A Review. Proc. Technol..

[2] Vautard F., Fioux P., Vidal L., Schultz J., Nardin M., Defoort B. (2011). Influence of the carbon fiber surface properties on interfacial adhesion in carbon fiber–acrylate composites cured by electron beam. Comp. Part A Appl. Sci. Manuf..

[3] Oliveux G., Dandy L.O., Leeke G.A. (2015). Current status of recycling of fibre reinforced polymers: Review of technologies, reuse and resulting properties. Prog. Mater. Sci..

[4] Xiao H., Lu Y., Zhao W., Qin X. (2014). The effect of heat treatment temperature and time on the microstructure and mechanical properties of PAN-based carbon fibers. J. Mater. Sci..

[5] Koumoulos E.P., Trompeta A.F., Santos R.M., Martins M., Monterio dos Santos C., Iglesias V., Böhm R., Gong G., Chiminelli A., Verpoest I. (2019). Research and Development in Carbon Fibres and Advanced High-Performance Composites Supply Chain in Europe: A Roadmap for Challenges and the Industrial Uptake. J. Comp. Sci..

[6] Dong-Kyu K., Kay-Hyeok A., Yun Hyuk B., Lee-Ku K., Sang-Yub O., Byung-Joo K. (2016). Effects of electrochemical oxidation of carbon fibers on interfacial shear strength using a micro-bond method. Carbon Lett..

[7] Almutairim M.K., Felemban R.A., Pasha S.E., Abo khashaba N.T., Mubaraki H.I., Yankesa R.A., Algamdi M.F., Bakkar A.M., Aldouweghri A.A., Sannan M.F. (2018). The Effect of Different Surface Treatments of Carbon Fibers and their Impact on Composites. Egypt. J. Hosp. Med..

[8] Gubanov A. (2015). Development of the Process of Electrochemical Modification of the Surface of Carbon Fibre with the Aim of Increasing the Strength of Carbon Fibre. Dissertation Thesis.

[9] Termine S., Trompeta A.F., Dragatogiannis D., Charitidis C.A. (2019). Novel CNTs grafting on carbon fibres through CVD: Investigation of epoxy matrix/fibre interface via nanoindentation. Matec Web Conf..

[10] Titchenal N., Lam H., Ye H., Gogosti Y., Ko F., Liu J., Willis P. SWNT and MWNT reinforced Carbon Nanocomposite Fibrils. Proceedings of the 45th Structural Dynamics & Materials Conference.

[11] Li D., Liu H., Chen B., Niu D., Lei B., Ye G., Jiang W., Shi Y., Yin L., Lai G. (2019). Amorphous Carbon-Induced Surface Defect Repair for Reinforcing the Mechanical Properties of Carbon Fiber. Materials.

[12] Moosburger-Will J., Jäger J., Strauch J., Bauer M., Strobl S., Linscheid F.F., Horn S. (2017). Interphase formation and fiber matrix adhesion in carbon fiber reinforced epoxy resin: Influence of carbon fiber surface chemistry. Compos. Interfaces.

[13] Di Salvo D.T., Sackett E.E., Johnston R.E., Thompson D., Andrews P., Bache M.R. (2015). Mechanical characterisation of a fibre reinforced oxide/oxide ceramic matrix composite. J. Eur. Ceram. Soc..

[14] Zhang J. (2012). Different Surface Treatments of Carbon Fibers and Their Influence on the Interfacial Properties of Carbon Fiber/Epoxy Composites. Ph.D. Thesis.

[15] (2010). Procedure for Strengthening Carbon Fibres. Patent.

[16] Georgiou P., Walton J., Simitzis J. (2010). Surface modification of pyrolyzed carbon fibres by cyclic voltammetry and their characterization with XPS and dye adsorption. Electrochim. Acta.

[17] Kim J., Mauchauffé R., Kim D., Kim J., Moon S.Y. (2020). Mechanism study of atmospheric-pressure plasma treatment of carbon fibre reinforced polymers for adhesion improvement. Surf. Coat. Technol..

[18] Hansong L., Yan Z., Na L., Xiaoran Z., Xiao H., Shuang L., Wenkuo L., Kai W., Shanyi D. (2019). Enhanced interfacial strength of carbon fiber/PEEK composites using a facile approach via PEI&ZIF-67 synergistic modification. J. Mater. Res. Technol..

[19] Liang Y., Li X., Semitekolos D., Charitidis C., Dong H. (2020). Enhanced properties of PAN-derived carbon fibres and resulting composites by active screen plasma surface functionalization. Plasma Process. Polym..

[20] Kainourgios P., Kartsonakis I.A., Dragatogiannis D.A., Koumoulos E.P., Goulis P., Charitidis C.A. (2017). Electrochemical surface functionalization of carbon fibres for chemical affinity improvement with epoxy resins. Appl. Surf. Sci..

[21] Li Z., Wang J., Tong Y., Xu L. (2012). Anodic Oxidation on Structural Evolution and Tensile Properties of Polyacrylonitrile Based Carbon Fibres with Different Surface Morphology. J. Mater. Sci. Technol..

[22] Bijwe J., Sharma M., Davim J.P. (2011). Carbon fabric-reinforced polymer composites and parameters controlling tribological performance. Wear of Advanced Materials.

[23] Theodoridou E., Besenhard J.O., Fritz H.P. (1981). Chemically modified carbon fibre electrodes: Part I. Bulk-functionalized carbon fibres. J. Electroanal. Chem..

[24] Jones C. (1993). Effects of Electrochemical and Plasma Treatments on Carbon Fibres Surfaces. Surf. Interf. Anal..

[25] Subramanian R.V., Jakubowski J.J. (1978). Electropolymerization on graphite fibers. Polym. Eng. Sci..

[26] Besenhard J.O., Jakob J., Möller P., Sauter R.F. (1989). Gic-Formation in Neutral Aqueous Electrolytes: An Undesired Side-Reaction during Electrochemical Surface Oxidation of Highly Oriented Carbon Fibres. Synth. Met..

[27] Chang J., Bell J.P., Shkolnik S. (1987). Electro-Copolymerisation of Acrylonitrile and Methyl Acrylate onto Graphite Fibres. J. Appl. Polym. Sci..

[28] Donnet J.B., Brendle M., Dhami T.L., Bahl O.P. (1986). Plasma treatment effect on the surface energy of carbon and CFs. Carbon.

[29] Sun M., Hu B., Wu Y., Tang Y., Huang W., Da Y. (1989). Surface of CFs continuously treated by cold plasma. Comp. Sci. Tech..

[30] Semitekolos D., Kainourgios P., Jones C., Rana A., Koumoulos E.P., Charitidis C.A. (2018). Advanced carbon fibre composites via poly methacrylic acid surface treatment; surface analysis and mechanical properties investigation. Comp. Part B Eng..

[31] Koumoulos E.P., Kainourgios P., Charitidis C.A. (2018). Assessing the integrity of CFRPs through nanomechanical mapping: The effect of CF surface modification. Matec Web Conf..

